# Cardiovascular Diseases and Marine Oils: A Focus on Omega-3 Polyunsaturated Fatty Acids and Polar Lipids

**DOI:** 10.3390/md21110549

**Published:** 2023-10-24

**Authors:** Cliodhna Caffrey, Anna Leamy, Ellen O’Sullivan, Ioannis Zabetakis, Ronan Lordan, Constantina Nasopoulou

**Affiliations:** 1 Department of Biological Sciences, University of Limerick, V94 T9PX Limerick, Ireland; c.caffrey7@universityofgalway.ie (C.C.); 19243839@studentmail.ul.ie (A.L.); ellenosullivan5@gmail.com (E.O.); ioannis.zabetakis@ul.ie (I.Z.); 2 Health Research Institute (HRI), University of Limerick, V94 T9PX Limerick, Ireland; 3 Bernal Institute, University of Limerick, V94 T9PX Limerick, Ireland; 4 Institute for Translational Medicine and Therapeutics, Perelman School of Medicine, University of Pennsylvania, Philadelphia, PA 19104, USA; ronan.lordan@pennmedicine.upenn.edu; 5 Department of Medicine, Perelman School of Medicine, University of Pennsylvania, Philadelphia, PA 19104, USA; 6 Department of Systems Pharmacology and Therapeutics, Perelman School of Medicine, University of Pennsylvania, Philadelphia, PA 19104, USA; 7 Laboratory of Food Chemistry—Technology and Quality of Food of Animal Origin, Department of Food Science and Nutrition, University of the Aegean, 814 00 Lemnos, Greece

**Keywords:** cardiovascular disease, omega-3 polyunsaturated fatty acids, polar lipids, cardiovascular risk, thrombosis, platelet-activating factor (PAF), eicosanoids, fish oils

## Abstract

Cardiovascular diseases (CVD) remain the leading cause of death across the globe, hence, establishing strategies to counteract CVD are imperative to reduce mortality and the burden on health systems. Dietary modification is an effective primary prevention strategy against CVD. Research regarding dietary supplementation has become increasingly popular. This review focuses on the current in vivo, in vitro, and epidemiological studies associated with that of omega-3 polyunsaturated fatty acids (n-3 PUFAs) and polar lipids (PLs) and how they play a role against CVD. Furthermore, this review focuses on the results of several major clinical trials examining n-3 PUFAs regarding both primary and secondary prevention of CVD. Notably, we place a lens on the REDUCE-IT and STRENGTH trials. Finally, supplementation of PLs has recently been suggested as a potential alternative avenue for the reduction of CVD incidence versus neutral forms of n-3 PUFAs. However, the clinical evidence for this argument is currently rather limited. Therefore, we draw on the current literature to suggest future clinical trials for PL supplementation. We conclude that despite conflicting evidence, future human trials must be completed to confirm whether PL supplementation may be more effective than n-3 PUFA supplementation to reduce cardiovascular risk.

## 1. Introduction

The burden of cardiovascular diseases (CVDs) has lessened over the last two decades due to the development of novel therapies; however, such diseases maintain their status as the leading cause of death globally [1]. CVD has been reported to account for 1 in 4 deaths across Europe, and 1 in 3 deaths in the United States [2,3]. Diet is known to be one of the most important risk factors for CVD prevention and treatment [4,5]. A wide range of other traditional risk factors are also associated with CVD, namely, smoking, obesity, and lack of physical activity. A maladaptive lifestyle characterized by these risk factors can contribute to an increase in oxidative stress and inflammation, contributing to metabolic dysfunction and atherogenesis over time [6]. 

Mechanistically, activated platelets play a major role in CVD [7,8,9]. Platelet activation, aggregation, and adhesion are all processes that contribute to the development of atherosclerosis over years, potentially leading to vessel occlusion, the rupture of atherosclerotic lesions, and thrombosis, causing myocardial infarction, stroke, or other complications [10]. 

Evidence suggests that the consumption of foods or supplements containing marine oils may affect chronic diseases and complications of metabolic dysfunction such as atherosclerosis [11,12]. These include omega-3 polyunsaturated fatty acids (n-3 PUFAs) and polar lipids (PLs). These lipids have been associated with the modulation of inflammatory and thrombotic pathways associated with atherogenesis [13]. 

The n-3 PUFAs are a heterogenous group of fatty acids naturally present in algae, fish, shellfish, and other marine sources that can be harvested to produce supplements and nutraceuticals [14,15]. In nature, n-3 PUFAs are most prevalent as neutral lipids (triglycerides, esters, etc.). However, they also present to a lesser extent in the form of polar lipids (PLs). PLs include glycerophospholipids, glycolipids, and sphingolipids. These molecules are characterized by a hydrophobic tail containing fatty acids and a polar head group. These characteristics mean that PLs are amphipathic. Consequently, PLs may increase the bioavailability of n-3 PUFA [16,17] attached to the polar head groups. These polar lipids are thought to be cardioprotective, but it is worth noting that some PLs may also exert cardioprotective effects independent of n-3 PUFA [18] as observed in non-marine PL extracts [19,20,21].

In this manuscript, we review published research, reviews, and the literature, to probe the role of n-3 PUFA- and PL-containing marine oils and their potential cardiovascular health benefits. We critically discuss crucial studies and trials that emerged from the literature, and we discuss the future of research in the field of marine oil cardioprotective products. In particular, we focus on the disparate results obtained in the REDUCE-IT and STRENGTH trials. Finally, we present a comparison of n-3 PUFA versus PL supplementation to identify evidence-based recommendations for conducting future clinical trials that may clarify and improve the current treatment and prevention strategies for both CVD and cardiovascular risk.

## 2. Methods

Manuscript record retrieval was completed using the following search terms: “cardiovascular disease” + “polar lipids”, “cardiovascular disease” + “omega-3 fatty acids”, “cardiovascular disease” + “omega-3 fatty acids” + “polar lipids”, “cardiovascular risk” + “omega-3 fatty acids”, “cardiovascular risk” + “polar lipids”, “cardiovascular risk” + “omega-3 fatty acids” + “polar lipids”, “inflammation” + “thrombosis” + “polar lipids”, “inflammation” + “thrombosis” + “omega-3 fatty acids”. All searches were completed using a combination of the Scopus, PubMed, and Web of Science databases from 1994 to 2023. The inclusion criteria encompassed original research articles and relevant reviews published in English between 1994 and 2023. Preference for inclusion was given to manuscripts that were closely aligned with the theme of this review article and published since 2010. Relevant literature cited in the identified literature were also considered for inclusion.

## 3. Marine Oils: Polyunsaturated Fatty Acids and Polar Lipids

In general, the consumption of supplements has increased over the past few decades due to increased consumer awareness and demand for wellness products [22]. Therefore, it is no surprise that the global nutraceuticals and supplements market was worth almost USD 353 billion in 2019 [23,24]. The consumption of marine oil supplements has steadily increased over the years due to their association with anti-inflammatory and cardioprotective effects relevant to public health [25]. Indeed, fish oils are the most commonly consumed dietary supplement aside from vitamin and mineral supplements in the US, whereby 7.8% and 1.1% of US adults and children respectively, consume fish oil supplements containing EPA, DHA, or a mix of n-3 PUFAs [26,27]. In 2022, fish oil consumption globally reached 3.6 million metric tonnes [28]. In contrast, the projected fish oil production in 2010 was estimated to be only 1 million metric tonnes [29]. This phenomenal growth is expected to continue with the fish oil market expected to expand further at a compound annual growth rate of 5.9% from 2022 to 2030, valued at USD 3.62 billion [28]. However, the added value of producing supplements means the value is considerably higher, with global sales of omega 3 supplements generating approximately USD 5.18 billion in 2019 alone [30]. While this growth is largely driven by human consumption, fish oils are also used in animal and pet foods, cosmetics, aquaculture, and pharmaceuticals [28,29,31]. However, there have been reports that n-3 PUFA supplements often do not contain the correct amount of n-3 PUFA or there is evidence of poor lipid quality or oxidation [32,33].

Despite their popularity among consumers, the scientific community is still at odds about the scientific evidence purporting cardioprotective effects in humans upon consumption. In this review, we discuss n-3 PUFA and PL marine oils and their potential cardiovascular effects. 

### 3.1. n-3 PUFA Structure and Function

**Figure 1:**
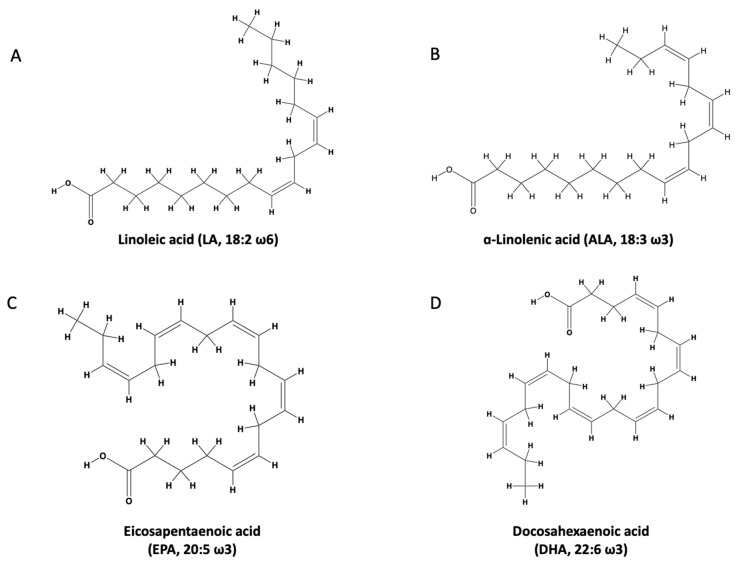
(**A**) linoleic acid; (**B**) alpha-linoleic acid; (**C**) eicosapentaenoic acid; (**D**) docosahexaenoic acid. Adapted with permission from [37].

The n-3 PUFAs have a double bond between the third and fourth carbon going from the end of the carbon chain (omega end), giving rise to the name n-3 PUFA. A short-chain n-3 PUFA is considered to have a chain that consists of 18 carbons or fewer. A long-chain n-3 PUFA has 20 or more carbons in its chain. Alpha-linolenic acid (ALA) is a common n-3 PUFA that is abundantly found in plant oils and the human diet, where it is found in soy, flaxseeds, and tree nuts in abundance (Figure 1). However, in marine oils the most abundant n-3 PUFAs are eicosapentaenoic acid (EPA) and docosahexaenoic acid (DHA). EPA is an n-3 PUFA that comprises a 20-carbon chain, making it a long-chain n-3 PUFA, with five *cis* double bonds. The double bonds can be found at carbons 5, 8, 11,14, and 17. Docosahexaenoic acid (DHA) possesses a 22-carbon chain. Its structure contains six cis-double bonds located at carbons 4, 7, 10, 13, 16, and 19 [34,35,36] (Figure 1). 

### 3.2. n-3 PUFA Cardiovascular Health Effects

The efficacy of n-3 PUFA treatment of CVD has long been controversial. The n-3 PUFAs are known to be an essential element of the platelet phospholipid membrane. Hence, they play a vital role in platelet function and are studied for their antiplatelet properties [38]. For over 20 years, supplementation of n-3 PUFA has been encouraged to curb the development of CVD [10]. While these supplements are voluntarily taken and prescribed for a wide range of medical conditions, they are predominately used for both the primary and secondary prevention of CVD [39]. 

**Figure 2:**
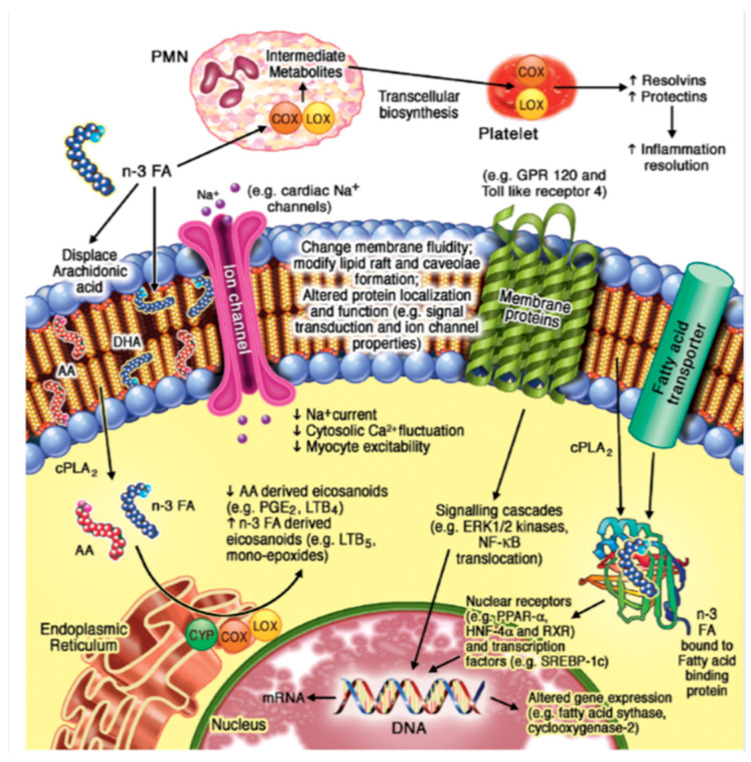
Hypothesized molecular effects of n-3 PUFAs on the cell membrane. Reproduced with permission [1].

The n-3 PUFAs are known to have the ability to alter cell structure and cell signaling by altering the configuration of lipids within the cell membrane (Figure 2) [40]. This has been demonstrated by several animal studies that report that the alteration of cellular function can occur by the addition of n-3 PUFAs via the diet [41,42]. Incorporation of n-3 PUFAs into the cell membrane can also modulate ion channels, such as L-type calcium (Ca^2+^) and sodium (Na^+^) [43]. In addition, n-3 PUFAs may directly associate with both proteins and membrane channels (Figure 2). An example of this can be observed from the direct modulation of the G protein-coupled receptor 120, or that of ion channels. Both actions have been noted to possibly aid in both anti-inflammatory and anti-arrhythmic responses associated with n-3 PUFAs, respectively [44]. Figure 2 highlights how both transcription factors and nuclear receptors contribute to the regulation of gene expression, which is of course a direct result of the addition of n-3 PUFAs. As a whole, n-3 PUFAs are known to act as natural ligands of numerous nuclear receptors within various tissues of the body, such as liver X receptors and retinoid X receptors. The interactions between such nuclear receptors and that of n-3 PUFAs are altered by cytoplasmic lipid binding proteins, which in turn can carry the fatty acids inside the nucleus. n-3 PUFAs also amend the role of transcription factors, for example, the sterol regulatory element binding protein-1c. This regulation in turn plays a part in inflammatory pathways [45]. Figure 2 also highlights the conversion of n-3 PUFAs from polar lipids in cell membranes to eicosanoids through three enzymes: lipoxygenase (LOX), cytochrome P450 (CYP450), and the cyclooxygenases (COX1 and COX2). Via incorporation into cell membranes, n-3 PUFA can supersede arachidonic acid (AA) and hence, this results in a reduction in AA-acquired eicosanoids [46]. This has been associated with a reduction in thrombosis, maladaptive vascular function, and inflammation. 

Another area of interest mechanistically has been the implication that n-3 PUFAs are required for the formation of specialized pro-resolving mediators (SPMs), involved in the so-called resolution of inflammation. This is a mechanism distinct from anti-inflammatory actions [47]. Such SPMs include protectins and resolvins, which are metabolites originating from the actions of the previously mentioned LOX and COX enzymes. It has been widely reported within animal models that n-3 PUFA-derived SPMs could possibly play a role in the reduction of chronic inflammation through the hypothesis of resolving inflammation [48]. However, evidence of these molecules exerting a beneficial effect in humans has been lacking [8,49] and the detection of resolvins in plasma and their functional relevance in human biology is an active but controversial field of research [50,51,52,53]. 

Both EPA and DHA appear to be the most functionally important n-3 PUFAs. Typically, they are both referred to as marine n-3 PUFAs due to their abundance in fatty (approx. 1–3.5 g/serving) and lean fish (approx. 0.1–0.3 g/serving), and other seafood. In addition, they are also found, although not equally, in various supplements. A summary of their relative concentrations of n-3 PUFAs can be found in Table 1. In addition, examples of pharmaceutical grade EPA and DHA used within the industry are also detailed.

**Figure 3:**
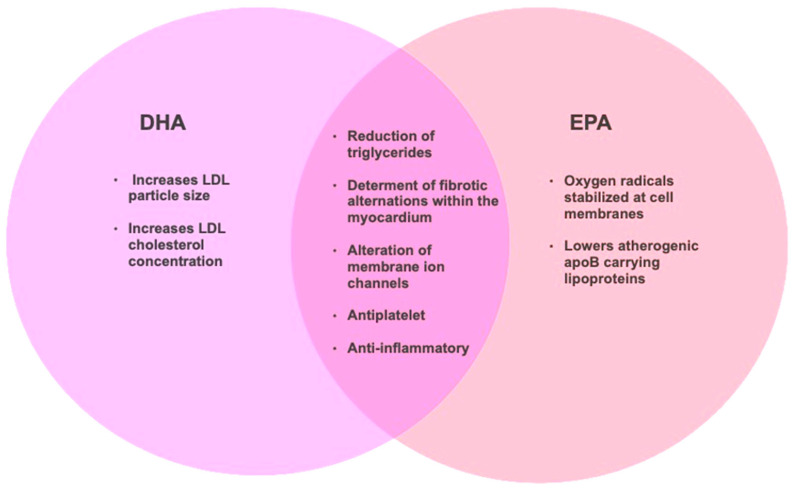
A summary of the potential benefits of DHA and EPA intake for cardiovascular health. Adapted with permission [62].

DHA and EPA exert a wide range of physiological effects, including the reduction in triglycerides, heart rate, blood pressure, and platelet aggregation [18,25]. Both n-3 PUFAs also enhance arterial compliance and flow-mediated dilation while also reducing pro-inflammatory cytokines and C-reactive protein (CRP) [55]. However, it has been consistently noted that such effects may be dependent on the specific health status or genetics of an individual [56,57,58], indicating that there may be a role for personalized nutrition and supplementation approaches [59]. EPA and DHA may also reduce plasma or serum concentrations of pro-inflammatory eicosanoids [60]. However, most research has focused on the use of EPA and DHA in combination, as opposed to their impact administered separately. EPA and DHA may exert differential effects on cardiovascular outcomes, particularly in lipid metabolism. Some of these effects, including the reduction in inflammation and oxidation are summarized in Figure 3. However, the link between EPA and DHA in the modulation of inflammation lipoprotein metabolism has yet to be confirmed. Hence, currently there is no clear advantage between DHA and EPA for the modulation of lipid metabolism. However, it is likely that a combination of both may yield the most advantageous health outcomes [61]. 

### 3.3. Polar Lipid Structure and Function

**Figure 4:**
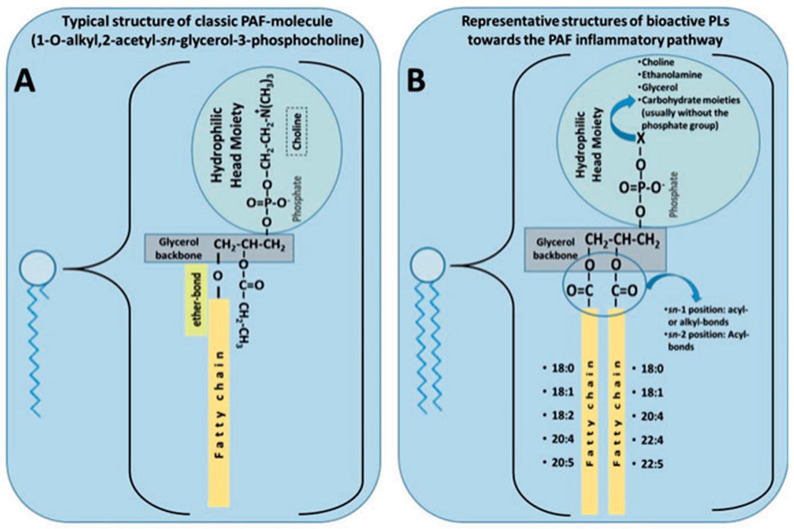
(**A**) The typical structure of a PAF molecule. (**B**) Representative structure of a bioactive polar lipid. Reproduced with permission [70].

While the previous section focused on the neutral forms of n-3 PUFAs, including ethyl esters, triglyceride, and fatty acid forms, some n-3 PUFAs are present as a constituent of PLs (Figure 4). Preliminary evidence from nutritional studies suggests that PLs with/without n-3 PUFAs in their structures may exert beneficial effects on CVD risk [63,64,65,66]. PLs are amphipathic molecules such as phospholipids or sphingolipids that are ubiquitous in nature. They are essential to the composition of cell membrane structure and function, cell signaling as secondary messengers, and lipid metabolism. They consist of a hydrophobic hydrocarbon tail and a polar hydrophilic head group [67]. Glycerophospholipids share a common assembly composed of a glycerol backbone attached to a phosphate group and two fatty acids esterified to the *sn-1* and *sn*-2 positions. At the *sn*-3 position, the head group is composed of a phosphate group and/or with phosphodiester linkages to organic molecules. These substituted head groups include choline (phosphatidylcholine), ethanolamine (phosphatidylethanolamine), serine (phosphatidylserine), or inositol (phosphatidylinositol). Sphingolipids replace the glycerol backbone with a sphingosine backbone, which is a long-chain amino alcohol that is amide-linked to the fatty acid and phosphate group [68,69]. Other common PLs include glycolipids and ceramides. 

### 3.4. Polar Lipids and Cardiovascular Health Effects

PLs are commonly found in foods such as olive oil, fish, meat, and dairy products associated with the Mediterranean diet [71,72]. The Mediterranean dietary pattern is strongly associated with a decreased risk of CVD [73] as demonstrated by the PREDIMED trials [74,75]. The Mediterranean diet has also been adopted outside of the Mediterranean region for the purpose of research, which appears to be a promising preventative and therapeutic option for CVD [76,77,78]. PLs are consumed in abundance as part of this dietary pattern. PLs have been postulated to be one of the constituents of the Mediterranean diet that may exert cardioprotective benefits via their antithrombotic and anti-inflammatory bioactivities against the actions of platelet-activating factor (PAF) and other inflammatory mediators [79,80,81,82]. PAF is a potent phospholipid mediator that interacts with its receptor (PAF-R) on the surface of numerous immune cells and platelets, causing platelet activation and pro-inflammatory cytokine release [83]. The production of PAF is stimulated by numerous cells such as platelets and leukocytes [84]. PAF is implicated in every stage of atherosclerosis through various mechanisms making it crucial to the process. The structure of PAF is characterized by an alkyl ether linkage, an acetyl group, and a phosphocholine group present at positions *sn*-1, *sn*-2, and *sn*-3 of the glycerol backbone, respectively [85]. PAF contributes to inflammation by mediating the adhesion of monocytes to the endothelium and in conjunction initiates gene transcription within monocytes resulting in the production of inflammatory cytokines. PAF generates an influx of Ca^2+^ ions, which increases endothelial permeability. This allows for the movement of LDL cholesterol and monocytes into the intima, allowing for the development of atherosclerotic plaque. Patients with CVD have elevated levels of PAF [84,86]. 

**Figure 5:**
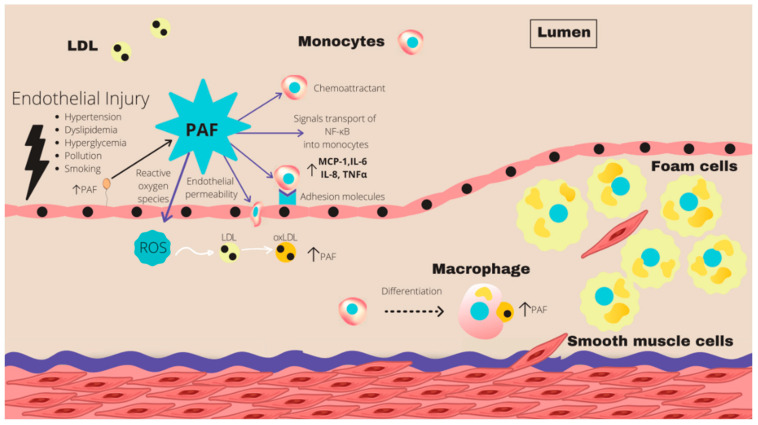
An illustration of the role of PAF in the initiation and progression of atherosclerotic plaque. Following exposure to injury, the endothelial cells are activated, triggering the synthesis of PAF and the expression of adhesion molecules, mediating the attachment of monocytes to the endothelium. PAF also triggers gene expression of pro-inflammatory cytokines such as IL-6 and TNF-α via NF-kB, and the production of ROS, which oxidizes LDL. PAF decreases the production of endothelial NO, increasing endothelial permeability. This allows for the movement of LDL and monocytes into the intima. PAF accounts for the polarization of monocytes into macrophages which engulf oxidized LDL, triggering the production of more PAF. Abbreviations: NF-kB, nuclear factor kB; IL, interleukin; PAF, platelet-activating factor; TNF-α, tumor necrosis factor α; LDL, low density lipoprotein; ROS, reactive oxygen species [87].

However, PAF is an important regulator of various physiological functions. If unregulated, it can result in a pro-inflammatory state leading to endothelial dysfunction and the development of atherosclerosis [71,83] (Figure 5). PAF and PAF-like molecules proceed via binding to a unique G protein-coupled receptor called PAF-receptor (PAF-R) [83]. PAF-R is expressed on platelets and is expressed by cells within the cardiovascular system. Ligand binding of PAF to the PAF-R provokes numerous intracellular signaling pathways which, if unregulated, can bring about a pro-inflammatory state, endothelial dysfunction, and the occurrence of atherosclerotic plaques [83]. 

Research suggests that PLs consumed in the diet are PAF antagonists that can inhibit PAF via their effects on the PAF receptor [83]. Indeed, some foods and natural products contain PAF antagonists [65]. This is due to the similarity in structure between PLs and PAF/PAF-like molecules, examples of which include phospholipids and sphingolipids [71], as can be seen in Figure 4. It has also been suggested that PLs can modulate the metabolism of PAF [79,88,89]. As this is a newer area of research, evidence supporting these claims are lacking in human trials to date, but research continues [85]. 

### 3.5. Implications of the Structural Differences between n-3 PUFAs and Polar Lipids

The n-3 PUFAs exist primarily esterified to triglycerides (neutral) or phospholipids, which are (polar) in nature. Hydrolysis causes n-3 PUFAs to exist as free fatty acids (neutral). Structurally, n-3 PUFA triglycerides differ to PLs as n-3 PUFAs comprise a glycerol backbone with three fatty acids attached to it. In contrast, PLs normally have two esterified fatty acids attached to the glycerol backbone as seen in Figure 4. PLs can form liposomes and micelles due to the differences in the physical–chemical [34]. PLs are an amphiphilic molecule, which means that PLs contain a hydrophobic tail and a hydrophilic head naturally. This gives rise to PLs to act spontaneously, as their hydrophilic region can navigate the aqueous phase and the hydrophobic region can navigate the non-aqueous phase, where it is functionally able to be soluble in fat [35]. On the other hand, n-3 PUFA triglycerides incur an exceedingly low water solubility, which may have a negative effect on the utilization of n-3 PUFA supplements [36]. As mentioned previously, PLs are found in the human diet as phospholipids and sphingolipids, which are essential components of biological membranes [35]. Whereas, n-3 PUFAs are found in the body as ALA, DHA, and EPA as previously mentioned. 

## 4. Marine Oils and Human Health

### 4.1. Cardioprotective Marine Oil Supplements Containing n-3 PUFA and Polar Lipids

Interest regarding marine oils grew from observations of the dietary patterns of Greenland Eskimos, who experienced a considerably lower incidence of cardiovascular disease attributed to their fatty fish-rich diet [54]. This has also been observed in Japanese populations, where on average one fish meal per day is consumed providing approximately 900 mg of n-3 PUFAs [90]. Research shows that consuming fatty fish as part of your weekly diet can significantly reduce the risk of CVD in comparison to a person that does not consume fish [91,92]. It is advised by the American Heart Association (AHA) to consume at least two meals containing fish per week. Fish consumption provides a wide array of dietary PUFAs both in neutral and PL form that are generally not as easily acquired via supplementation [93]. Furthermore, there are additional benefits associated with consumption of the whole fish, including the addition of vitamins, minerals, and proteins. However, the AHA has recommended n-3 PUFA supplements if fresh fish is unavailable to meet recommended n-3 PUFA requirements and to reduce CVD risk [94,95]. However, the evidence regarding n-3 PUFA supplementation is not as straightforward and there are some inconsistencies regarding their role in both primary and secondary prevention of CVD. Indeed, large trials and meta-analyses have yielded inconsistent findings [96,97].

### 4.2. n-3 PUFA in Clinical Trials

Early trials conducted examining n-3 PUFA consumption focused on cardiovascular diseases, which largely concluded that n-3 PUFA were efficacious in the treatment and prevention of CVD. Therefore, there was general support for their consumption [98]. Examples of older trials that generally supported n-3 PUFA consumption to improve CVD risk include the Diet and Reinfarction Trial (DART), the Lyon Heart Study, and the Gruppo Italiano per lo Studio della Sopravvivenza nell’Infarto miocardico Prevenzione trial (GISSI-P) [99,100]. However, due to limitations in these studies such as small sample sizes, the findings of these trials are often dismissed when examining the effects of n-3 PUFAs on CVD. With advances in cardiovascular knowledge, the results of many more recent randomized controlled trials (RCTs) have challenged previously recorded data [101,102]. More recently published studies are less encouraging regarding the importance of n-3 PUFAs and a reduction in CVD [102], and many studies are now focusing on alternative approaches including the delivery of n-3 PUFAs in other forms such as PLs [18].

In recent years, there have been several large-scale trials that have examined the efficacy of n-3 PUFA supplementation. These include the REDUCE-IT trial and the STRENGTH trial. These trials were touted as the studies that may end the debate regarding n-3 PUFAs and their cardioprotective effects. Therefore, in Section 4.2.1 and Section 4.2.2. we discuss the outcomes, strengths, and limitations of these trials and focus on how these studies have contributed to our growing knowledge regarding n-3 PUFA supplementation, cardiovascular health, and clinical trials. 

#### 4.2.1. The REDUCE-IT Trial in Context

Icosapent ethyl (IPE), also known AMR101 or commercially as Vascepa^®^, is produced and marketed by the Irish company Amarin Pharma. IPE is a supplement composed of highly purified EPA. The product was initially approved by the United States Food and Drug Administration (FDA) for the treatment of hypertriglyceridemia [103]. The reduction in cardiovascular events with the icosapent ethyl intervention trial (REDUCE-IT) was established to determine the potential of IPE to reduce ischemic events in patients diagnosed with cardiovascular disease [104]. This was a major multicenter, double-blinded, randomized, placebo-controlled trial (mineral oil), which caused controversy between scientists and health experts since its publication [103]. Bhatt and colleagues enrolled over 8000 patients with established cardiovascular disease or elevated risk, of which over 70% had experienced a previous cardiovascular event [105]. Participants were enrolled to the REDUCE-IT study if they were ≥45 years of age with previous CVD or if they were ≥50 years of age with diabetes and at least one other risk factor. These may include elevated fasting LDL levels, triglyceride levels, or patients receiving statin therapy. Patients were followed for a median of 4.9 years. The primary composite end points were cardiovascular mortality, nonfatal stroke, nonfatal myocardial infarction, unstable angina, or coronary revascularization. 

Since the majority of the study population enrolled had established CVD, this study is generally viewed as a secondary prevention of CVD with n-3 PUFA supplementation study [106]. The results of the trial revealed that consumption of 2 g IPE *po bid* (4 g total per day) reduced the risk of ischemic cardiovascular events and death. Of those assigned to the IPE group, a primary endpoint occurred in 17.2%, versus 22.0% in the placebo group (*p* < 0.001; hazard ratio, 0.75; 95% confidence interval [CI], 0.068–0.83) or an absolute difference of 4.8%, irrespective of triglyceride levels at baseline or during the study [105]. Additional analyses supported that IPE supplementation may reduce CVD risk relating to high triglyceride levels [107]. Overall, IPE supplementation appeared to be safe with limited side effects including more frequent nonfatal adverse bleeding events, and more frequent hospitalization for peripheral edema and atrial fibrillation in the IPE group versus the placebo group [105,106].

While these widely anticipated results were well received initially, with the study being described as rounding the corner on residual risk [108], several concerns were raised regarding the study design. On closer inspection, it was noted that the placebo mineral oil used for the trial was not inert, and that this may in fact have increased the placebo groups’ risk for cardiovascular events. Indeed, the mineral oil intake was associated with an increase in LDL-C (7.4%), CRP (37.6%), and apolipoprotein B (6.7%) [105]. Similar increases in these biomarkers were reported as a consequence of mineral oil ingestion were previously reported in the ANCHOR [109] and MARINE [110] trials, which also investigated the use of IPE for cardiovascular risk reduction. In these studies, it is possible that the differences in the apparent reduction of cardiovascular risk associated with IPE treatment may be explained by the increased risk of exposure to mineral oil in the placebo group [103]. However, this is disputed in a review published by the REDUCE-IT trial authors [111]. Indeed, independent reviews by the FDA and other health agencies (Canada Health and the European Medicines Agency) concluded that the increases in these cardiovascular biomarkers associated with mineral oil may only partially explain the major cardiovascular events reported between the two randomized groups [103].

The data have not become any clearer since the trial was published. A meta-analysis of thirteen randomized controlled trials conducted by Hu et al. in 2019 concluded that consumption of marine n-3 PUFA supplementation does indeed lower the risk for myocardial infarction, both CHD total and death and also for both CVD total and death [112]. This meta-analysis also calculated the reduced risk excluding the REDUCE-IT trial due to the controversary surrounding its findings and still deduced that n-3 PUFA consumption was inversely associated with CVD. However, there were limitations to this study such as being unable to conduct subgroup analysis due to lack of study-level data available and the author does state that there is a need for additional large trials, particularly those undertaken using high doses of n-3 PUFA supplementation to confirm and extend these findings. Another meta-analysis, which was conducted by Shen et al. in 2022, found that additional n-3 PUFA supplementation may decrease the risk for incidence of major adverse cardiovascular events, cardiovascular death, and myocardial infarction [113]. However, the study also deduced that n-3 PUFA did not significantly impact all-cause death, stroke, and revascularization. The study did, however, have minor limitations such as some subgroups containing a relatively low number of studies and more research is likely required to support and validate these findings.

In contrast, numerous studies failed to support the positive findings of the REDUCE-IT, JELIS, GISSI-P, and GISSI-HF (heart failure) trials [99,105,114,115]. These include trials such as VITAL (The VITamin D and OmegA-3 triaL), ORIGIN (Outcome Reduction with an Initial Glargine Intervention trial) and ASCEND (A Study of Cardiovascular Events in Diabetes) [116,117,118]. Collectively, these trials do not support the use of n-3 PUFA supplementation for cardioprotection against CVD. However, these trials differ in various aspects such as the placebo used, entry criteria, and the dosage of n-3 PUFA administered, which may account for the differences in the findings between these studies. Comparisons between some of these studies are presented in Table 2. 

#### 4.2.2. The STRENGTH Trial in Context

Epanova^®^ was originally produced by Omthera Pharmaceuticals Inc. in New Jersey, USA before being acquired by AstraZeneca. Epanova^®^ is a 1 g supplement that delivers 850 mg of n-3 PUFA in the form of carboxylic acids. In the production process, the n-3 PUFA are hydrolyzed and distilled from ethyl esters into PUFA carboxylic acids. The final concentration of EPA and DHA in this drug is 75%. The aim of this therapeutic was to maximize the EPA and DHA bioavailability for the treatment of hypertriglyceridemia. Epanova^®^ does not need to be hydrolyzed by lipases from the pancreas, allowing easier absorption by the intestines and eliminating the need for consumption with a high-fat meal [123,124]. The STRENGTH trial was designed to examine the effects of Epanova^®^ on reducing rates of cardiovascular events in statin-treated patients with hypertriglyceridemia [124]. STRENGTH involved a randomized, placebo-controlled, double-blind study between 13,078 patients in a 1:1 ratio treatment of 4 g/day of n-3 PUFA carboxylic acids (Epanova^®^) versus 4 g/day of a corn oil placebo. Participants were 62.5 years old, consisting of 35% female participants. A corn oil placebo was chosen for this trial over mineral oil and liquid paraffin because it has a reduced incidence of gastrointestinal adverse effects and provides adequate calorie management. The criteria to participate in the STRENGTH trial included patients with high cardiovascular risk (CVR), determined atherosclerotic cardiovascular disease (ASCVD), established diabetes with an addition risk factor, or other high-risk primary prevention patients based on risk factor assessments and age factors, triglyceride levels ranging between 180 and 500 mg/L, and high density-lipoprotein cholesterol (HDL-C) levels of <42 mg/dL (men) or <47 mg/dL (women) [121]. Additionally, participants were required to be on 100% statin therapy four weeks prior to the trial’s commencement date and low-density lipoprotein cholesterol (LDL-C) levels had to be <100 mg/dL [121,124]. 

The primary endpoints of the trial were the composite of cardiovascular mortality, nonfatal myocardial infarction, nonfatal stroke, unstable angina, and revascularization [121]. An interim analysis led to the early termination of the trial due to a perceived low clinical benefit of treatment versus the placebo. There were 1384 validated initial primary endpoint occurrences out of the predicted 1600 primary events among the almost 13,000 patients who completed the trial. In total, the primary endpoint occurred in 12% of the treated cohort (*n* = 785) versus 12.2% (*n* = 795) of the corn oil cohort. Furthermore, gastrointestinal adverse events occurred more frequently in the Epanova^®^ group versus the corn oil cohort (24.7% versus 14.7% respectively). Likewise, atrial fibrillation was more frequently observed in the Epanova^®^ group compared to the corn oil group (2.2% versus 1.3%) [121]. 

The addition of n-3 PUFA carboxylic acids to individuals on statin therapy with high cardiovascular risk compared to those on corn oil resulted in no meaningful change in a composite outcome of major adverse cardiovascular events. Therefore, the data reported do not support the use of these n-3 PUFAs to reduce cardiovascular risk. 

### 4.3. What Can We Learn from the STRENGTH and REDUCE-IT Trials

The role of n-3 PUFA supplementation and heart health strikes up great controversy due to heterogeneity between different clinical trials. This is clearly evident among some of the largest clinical trials. The most obvious examples include the more recent apparent successful reduction in cardiovascular risk observed in the REDUCE-IT trial and the apparent failure to reduce cardiovascular risk observed in the STRENGTH trial [125]. Some of these differences are presented in Table 3. There are several reasons why the results of these two important trials may differ. 

To begin with, both trials opted to use different formulations of n-3 PUFAs, as was previously alluded to. The REDUCE-IT trial provided participants with a 2 g dose of IPE (Vascepa) po bid or an equivalent style placebo containing mineral oil; participants were on a medically controlled 100% statin treatment [104]. Whereas the STRENGTH trial provided participants with Epanova^®^, which is a 1 g supplement that delivers 850 mg of n-3 PUFAs in the form of carboxylic acids versus a corn oil placebo. It is clear that the type, form, and dosing of the n-3 PUFAs differed between the trials. Another major difference is the absorption of the two products. IPE needs to be converted in the liver by hepatic conversion. In contrast, Epanova^®^ is a carboxylic acid that has been exposed to additional manufacturing processes that allows the product to be consumed without the requirement of further hydrolyzation by pancreatic lipases [124]. This posed the question of whether differing levels of bioavailability were at play. Indeed, higher serum EPA levels were measured in the REDUCE-IT cohort (144 μg/mL) versus the STRENGTH cohort (89 μg/mL) [126]. This is one potential reason for the observed disparity in findings between the trials. It was also questioned whether DHA may pose some harm in the STRENGTH trial, thus explaining the differing outcomes. However, a secondary analysis of the STRENGTH cohort indicated that there was no significant increase in benefit or any adverse outcomes in individuals with the highest levels of serum EPA or DHA [127].

As aforementioned, it is also important to acknowledge that the placebos used in both trials were different from each other, and this may indeed affect outcomes due to the potential negative effects of mineral oil on cardiovascular health [103,126]. Therefore, using mineral oil as a placebo may affect trial outcomes and raise the cardiovascular risk of those in the placebo group, falsely indicating a beneficial effect in the treatment group. However, these arguments are still being debated [103]. A cohort study using patients from the Copenhagen General Population Study (CGPS) was conducted to mimic the trial design of both studies to explain differences in observed CRP and serum lipid levels [128]. Patients who met the inclusion criteria took part in trial designs that emulated both the STRENGTH (*n* = 6862) and REDUCE-IT (*n* = 5684) studies. The authors of this study concluded that the contrasting results of both trials were likely due to a difference in the effect of the placebo oil used and not of the treatments assessed, as the mineral oil increased serum lipids and CRP [128]. However, this only partly explains the perceived benefit seen in the REDUCE-IT trial. Approximately, an additional 13% risk reduction may be due to a potential benefit of IPE, chance, or other factors [126]. Another way that both trials differed is in their enrollment criteria. While both trials needed patients with elevated lipid levels for study admission, REDUCE-IT only required mild hypertriglyceridemia (135–499 mg/dL) [105], whereas the STRENGTH trial required triglyceride levels between 180 and 500 mg/dL [121]. While minor, differences in enrollment may bias trial outcomes. 

Several n-3 PUFA products on the market are generally recognized as safe (GRAS). However, both trials indicated that there was an increased incidence of atrial fibrillation among participants [126]. Therefore, at a population level, it is important that incidence of atrial fibrillation is continually monitored. 

### 4.4. Marine Oil Polar Lipids: Innovations and Human Health

The majority of fish oil products on the market are neutral n-3 PUFA products. PL products are less frequently available due to the loss of PLs during the degumming processes conducted in industrial production of fish oil [129]. Although n-3 PUFAs have been extensively studied for their potential health benefits, particularly in terms of CVD, PLs may be more effective as carriers of n-3 PUFAs due to their increased bioavailability [16,18,129]. Krill oil is an example of a product that contains a high proportion of n-3 PUFAs bound to phospholipids [130,131]. The 72-hour bioavailability of 700 mg DHA with EPA in krill oil was assessed in comparison to that of fish oil and krill meal within a randomized trial containing 15 healthy participants. In this study, when considering the primary endpoint, DHA along with EPA contained increased bioavailability in the krill oil sample compared to that of fish oil and krill meal. However, in terms of secondary endpoint, the results were conflicting. The bioavailability of the samples did not differ, which suggests that the phospholipids were not absorbed any better than that of the triglycerides [132,133]. Hence, further studies are ultimately required to confirm this hypothesis. In addition, a study undertaken by Lapointe, et al. [134] concluded that the bioavailability of the sample containing DHA and EPA in the form of that of PL esters was greater than that of the cohort containing n-3 PUFAs in the ethyl ester form.

Although not an extensive list, several studies in Table 4 indicate that marine PLs may counteract thrombosis and inflammation [18,135]. One study showed that oil extracts from fish such as sea bass, plaice, coley herring, and rainbow and golden trout exhibited antiaggregatory properties against PAF-induced rabbit platelet aggregation in vitro [136]. All six fish are widely consumed in Europe. In a more recent study, fish oil obtained from salmon, herring, and boarfish, along with their processing by-products exhibited antithrombotic effects, against PAF and thrombin-induced human platelet aggregation due to their polar lipid content in vitro. Indeed, neutral lipids from the same fish did not exhibit the same level of antiplatelet activity despite their n-3 PUFA compositions [137]. Similar studies in human platelet aggregation studies against PAF and thrombin in vitro with salmon polar lipids [138] and food-grade salmon polar lipids [139] have shown that marine oils rich in PLs may exert favorable antiplatelet effects.

Fish fatty acid composition can change due to a variety of factors [140], and many researchers have shown that fish oil compositions change in response to diet alterations [141,142]. Food processing by-products are often used in animal feed. One such by-product is olive pomace (OP), which exhibited anti-PAF effects in vitro [143,144]. In one study, both sea bass (*Dicentrachus labrax*), and gilthead sea bream (*Sparus aurata*) were fed diets containing OP [145]. The results of this study indicated the PLs of the gilthead sea bream consisted of PAF inhibitors known to inhibit PAF both in vivo and in vitro likely accruing to a great extent due to the OP feed. However, incorporation of OP within fish feed at 8% appeared to negatively affect mortality and growth rate within sea bass, but a 4% OP diet was more tolerable. Oils obtained from these fish exhibited antiplatelet actions against PAF in vitro. To determine what lipids were responsible for the observed activity, Nasopoulou, et al. [146] isolated a number of lipid fractions to elucidate the structures and biological activity of the PLs purported to be responsible for the cardioprotective activity observed in vitro. Seven lipid fractions extracted from the fish that consumed the OP diet exhibited potent inhibitory actions against PAF-induced platelet aggregation, in comparison with that of those fed with the conventional fish oil (FO) diet. Moreover, the balance of PL fractions of fish, which were consuming the OP diet resulted in a large increase in inhibitory activity against platelet aggregation as opposed to their respective PL fractions obtained from fish fed the FO diet. This likely suggests that antiplatelet properties of the OP were likely increased in the fish flesh and oils through the OP diet. Indeed, when the OP-fed gilthead seabream (0.06%) fish oil was fed to hypercholesterolemic rabbits, a reduction in plaque size was observed versus the cholesterol diet (1%) control rabbits, indicating a potential anti-atherosclerotic effect of the fish PL [147]. These affects may also in part be due to the observed modulation of PAF metabolic enzymes including PAF-acetylhydrolase (PAF-AH) both in vitro and in vivo [147,148]. 

When assessed in healthy human volunteers, OP-fed fish consumption did not significantly affect multiple cardiovascular markers with the exception of an elevated PAF-CPT (1-alkyl-2-acetyl-sn-glycerol-choline-phosphotransferase) and reduced arachidonic acid levels in red blood cells [149]. However, this study is still rather promising considering this was a healthy population. Further studies in patients with higher CVD risk may indicate whether consumption of such functional foods may benefit patient cardiovascular health. Collectively, these studies further highlight the role that both PAF and its metabolism play in atherosclerosis and the role that future fish PL-based therapeutics may play in the battle against CVD. Indeed, multiple studies have demonstrated potential antiplatelet properties of fish oil PLs in vitro against PAF and various platelet agonists [137,138,139,150,151,152] and in various models of CVD in vivo [145,153]. However, it should be noted that PL sources characterized by lower levels of n-3 PUFAs such as dairy and meat also exhibit antiplatelet effects to a similar extent [21,154,155], indicating the promise of developing PL-based therapeutics generally.

**Table 4:** An overview of some of the studies investigating marine polar lipids possessing antiplatelet and anti-inflammatory activities in vitro and in vivo. While there has been much advancement in this field, further research is required.

Marine Lipid Sources	Experiments Conducted	Results	Reference
Salmon fillet (*Salmo salar*)	Investigation of the in vitro inhibition by salmon PL extract against PAF and thrombin-induced platelet aggregation in human PRP.	Salmon PL, TNL, and TL fractions from PE and PC showed high inhibitory activity against PAF and thrombin-induced platelet aggregation. These fractions had high concentrations of n-3 PUFAs.	[138]
Salmon fillet (*Salmo salar*)	Examination of the antiplatelet effects of raw and cooked salmon fillet PLs using different techniques against PAF-, thrombin-, collagen-, and ADP-induced platelet aggregation in human PRP.	All PL extracts exhibited potent antiplatelet effects. The extract was abundant in n-3 PUFAs.	[156]
Salmon fillet (*Salmo salar*)	Investigation of the in vitro inhibition by salmon food grade PL extracts against PAF- and thrombin-induced platelet aggregation in human PRP.	Food grade salmon extracts inhibited both PAF- and thrombin-induced platelet aggregation. The extract was abundant in n-3 PUFAs.	[139]
Salmon, herring, and boarfish by-products (*Salmo salar, Clupea harengus*, and *Capros aper*)	Examination of the in vitro inhibition of PAF-, thrombin-, collagen-, and ADP-induced platelet aggregation in human PRP by fish by-products isolated from salmon, herring, and boarfish.	All PL extracts were abundant in n-3 PUFAs and exhibited potent antiplatelet effects against various platelet agonists.	[137]
Salmon PL extract (*Salmo salar*)	Assessment of the antineuroinflammatory actions of salmon PLs in cell culture.	Salmon PLs demonstrated potential anti-inflammatory and antioxidant actions DI TNC1 rat astrocytes stimulated with amyloid-beta or LPS as a control by downregulating PAF receptor expression and reducing oxidative stress.	[157]
Sardines and cod liver oil (*Sardina pilchardus* and *Gadus morhua*)	Investigation of the antiplatelet in vitro properties of TL, TNL, and TPL in WRP.	TPL strongly inhibited PAF-induced platelet aggregation.	[151,158]
Sea bream and sea bass (*Sparus aurata* and *Dicentrarchus labrax*)	Investigation of the in vitro antiplatelet properties of TL, TNL, and TPL in WRP.	Inhibition of PAF-induced WRP aggregation.	[159]
Sea bream and sea bass (*Sparus aurata* and *Dicentrarchus labrax*)	Assessment of the anti-atherogenic effects of PL consumption in 12 male hypercholesterolemic rabbits versus a control group not receiving PL.	The PL-enriched diet modulated PAF metabolism and reduced circulatory PAF levels, which may be linked to a reduction in atherosclerotic plaques in these rabbits.	[145,147]
Dulse (*Palmaria palmata*)	Assessment of dulse PL and their inhibitory effects versus LPS-induced NO production.	PLs downregulated iNOS activity demonstrating anti-inflammatory properties.	[160]
Various algae-derived lipids (*Chondrus crispus*, *Palmaria palmata, Porphyra dioica*, *Pavlova lutheri*)	Various algae-derived lipids were assessed for anti-inflammatory activity in LPS-stimulated THP-1 macrophages in cell culture.	All lipids exhibited anti-inflammatory activity via mediating toll-like receptors, chemokines, and NF-κB.	[161]
Fresh and fried cod (*Gadus morhua*)	Test the PAF-like and anti-PAF properties of lipid fractions of fresh and fried cod, against PAF-induced platelet aggregation in WRP.	Lipid fractions (TPL and TNL) from fried and fresh cod showed inhibitory activity as well as slight platelet aggregation, indicating presence of both PAF agonists and inhibitors.	[162]

Abbreviations: ADP, adenosine diphosphate; iNOS, inducible nitric oxide synthase; LPS, liposaccharide; n-3 PUFAs, omega-3 polyunsaturated fatty acids; NF-κB, nuclear factor kappa B; NO, nitric oxide; PAF, platelet-activating factor; PC, phosphatidylcholine; PE, phosphatidylethanolamine; PL, polar lipids; THP-1, acute monocytic leukemia cell line; TL, total lipids; TNL, total neutral lipids; TPL, total polar lipids; WRP, washed rabbit platelets.

A significant proportion of the n-3 PUFA composition of fish is obtained through dietary sources including microalgae, phytoplankton, and cyanobacteria [163]. Therefore, microalgae are becoming increasingly popular as a source of high-value compounds with interesting bioactivity and chemical diversity. That said, the knowledge and understanding around their PLs’ characteristics remains largely limited [164]. Algae contain lipids such as n-3 PUFAs with antioxidant potential [161,165], which are sometimes attributed to the presence of glycolipids that are also known to exhibit antitumor and anti-inflammatory properties [166,167]. Indeed, it has been suggested to bypass the extraction of fish oil entirely and to instead focus on the production of n-3 PUFA supplements and nutraceuticals from microalgae and macroalgae, as they are a source of high-value lipids. Moreover, recent studies have suggested there is an abundance of therapeutic and pharmacological potential in relation to *Spirulina* biomass. Strong in vitro anti-thrombin and anti-PAF activities have been reported for extracts containing n-3 PUFA-rich PL fractions of *Spirulina subsalsa* [168] and *Chlorococcum* sp. [169]. Macroalgae are also under investigation for their PL composition [170,171]. Both *Palmaria palmata* and *Grateloupia turuturu* are rich sources of EPA *Palmaria palmata*, and PL extracts from these macroalgae exhibit antioxidant effect [172,173]. 

Other exciting marine sources for PL include sea urchins [174]. Lipids from the edible gonads of the sea urchin (uni) have been extensively studied [175]. Furthermore, there is the potential to use other parts of the sea urchin for the development of novel lipid-based products. For example, the sea urchin body wall, dermis, and epidermis of the endoskeleton, are thought to inhibit MAPK p38, COX-1, and COX-2, indicating potential anti-inflammatory effects [176]. Indeed, other sea creatures including tunics like *Halocynthia aurantium* [177] appear to harbor PLs with potential cardioprotective effects. The vast array of creatures in the oceans that contain abundant and novel PLs means that there is a vast area of PL research yet to be explored. 

Another area of research that has gained attention is the formulation of oils that use combinations of fish oils with oils from other sources, including plant extracts like chamomile oil, schisandra oil, or motherwort oil. One study showed that the immunomodulatory and antioxidant capacity of fish oil was improved when combined with chamomile and schisandra oil in vitro and in vivo, indicating potential synergistic effects of the fixed combination of oils [178]. This is a relatively underexplored area of research regarding fish PLs, which warrants further investment in research.

Despite all of these promising areas of research, further investigation is required to establish many of the PL-related findings in vivo. More clinical trials are also required to further investigate PLs and their effects on cardiovascular health. There are limited examples of PLs used for treatment of human conditions. However, although not related to CVD, a PL-rich pulmonary surfactant known as poractant alfa has been used in Russia and elsewhere to treat premature neonates with respiratory distress syndrome in combination with standard therapies [179], indicating that there is certainly scope for such products to be brought to market. 

Lastly, studies investigating marine PLs use a variety of lipid sources and isolation methods to bioprospect for a variety of potential bioactivities that may be beneficial for human health. These have been extensively reviewed [16,67,180]. However, the majority of these studies have been conducted using non-food-grade solvents that are toxic for human consumption, which, even if evaporated, may leave residues that are potentially dangerous in the oils. Some studies have investigated the use of food-grade extraction protocols in marine and non-marine lipids sources and noted differences in biological activity between conventional extraction methods and food-grade extraction methods for PL extracts due to differences in product composition [139,181,182]. Therefore, it is important that future studies consider the use of food-grade solvent extraction procedures when bioprospecting for potential bioactives in novel sources to ensure that such products may be safely evaluated in vivo. Indeed, it may also be worth considering the evaluation of such products using simulated gastrointestinal digestion (SGID) protocols also. 

## 5. Conclusions and Future Research Directions

In this review, we investigated the evidence surrounding marine oil consumption and cardiovascular health. In particular, we focused on n-3 PUFA and PL supplementation and their capacity to reduce cardiovascular risk. In the n-3 PUFA research space, many large clinical trials have been conducted with variable results because of differing trial design, placebos used, doses, and the form of n-3 PUFA consumed. An in-depth review of the REDUCE-IT and STRENGTH trials was conducted. Generally, while n-3 PUFAs may provide some cardiovascular benefits, large-scale trials have failed to conclusively support their use for cardiovascular risk reduction. This is largely due to differences in trial design, placebo use, and the different forms of n-3 PUFAs that have been assessed. The consumption of n-3 PUFA supplements is high worldwide but likely poses limited risk for adverse events. Trials largely expressed concerns about the increased incidence of atrial fibrillation, which should be monitored closely at a population level. This review also evaluated the role and potential of n-3 PUFAs withing dietary PLs and their potential cardiovascular benefits for risk reduction, through the examination of both in vitro and in vivo studies. Evidence regarding PL supplementation, although promising, is limited and further research is required. Given the large gaps within the literature remaining for both n-3 PUFAs and PLs, it is difficult to draw concrete conclusions. In designing future studies, we suggest that the form of n-3 PUFA used needs to be taken into account along with the choice of placebo. Studies investigating PL forms of n-3 PUFAs are also warranted in humans to determine whether the polar head group conveys greater bioavailability of n-3 PUFAs, thus increasing their efficacy and potency.

## Figures and Tables

**Table 1:** A summary of EPA and DHA concentrations in various n-3 PUFA supplements. Data adapted with permission [54].

Supplements	n-3 PUFA Content Per Gram of Oil
Krill oil	205 mg
Tuna oil	460 mg
Fish oil (standard)	300 mg
Cod liver oil	200 mg
Algal oil	400 mg
**Pharmaceuticals**	**EPA/DHA content per gram of oil**
Omacor^®^ (ethyl esters)	460 mg (EPA) and 380 mg (DHA)
Epanova^®^ (carboxylic acids)	550 mg (EPA) and 200 mg (DHA)
Vascepa^®^ (ethyl ester)	900 mg EPA

**Table 2:** Summary of investigations focusing on the effects of n-3 PUFAs on CVD in both healthy and high-risk patients.

Trial	*N*	Age	Formulation and Dose	Inclusion Criteria/Cohort Characteristics	Duration (Years)	Placebo
Successful—Primary endpoint reached *						
REDUCE-IT [105]	8179	45 with CVD or 50 with DM	IPE 4 g	Patients with established CVD or DM on statin therapy with increased TG levels	4.9	Mineral oil
EVAPORATE [119]	80	30–85	IPE 4 g	Patients with confirmed coronary artery stenosis on statin therapy with increased TG levels.	1.5	Mineral oil
JELIS [114]	18,645	Men 40–75 Women up to 75 years	EPA 1.8 g +pravastatin or simvastatin	Patients with previous MI or PCI or with confirmed angina pectoris or without CVD.	4.6	No placebo
CHERRY [120]	193	68 ± 10	Pivastatin + EPA 4 mg + 1800 mg	Patients with CHD after PCI	6–8 months	Pitavastatin 4 mg/day
Unsuccessful—Failed to reach primary endpoint *						
STRENGTH [121]	13,078	18–99 (>40 for men 50 for women if with DM)	EPA + DHA carboxylic acids. 4 g	LDL-C < 100 mg/dL, on statins, TG levels 180–499 mg/dL, HDL-C < 42 mg/dL in men, <47 mg/dL in women, patients with CVD or diabetes with risk factors.	5	Corn oil
VITAL [116]	25,871	Men > 50 Women > 55	EPA + DHA 1 g	Healthy men > 50 and healthy women > 55. TG levels not specified.	5.3	Not specified
ASCEND [118]	15,480	>40	EPA + DHA 1 g	Persons older than 40 years with DM without CVD.	7.4	Olive oil 1 g
ORIGIN [117]	12,536	50	EPA + DHA 465 mg + 375 mg	High risk of CVD + impaired fasting glucose/glucose intolerance/DM.	6.2	Olive oil 1 g
OMEMI [122]	1027	70–82 + Recent (2–8 weeks) MI	EPA + DHA 930 mg + 660 mg	Recent acute MI	2	Corn oil

* According to the study authors. Abbreviations: IPE: icosapent ethyl, DM: diabetes mellitus, TG: triglyceride, MI: myocardial infarction, PCI: percutaneous coronary intervention.

**Table 3:** Comparisons between the STRENGTH and REDUCE-IT trials.

Clinical Trial	STRENGTH	REDUCE-IT
Number of participants	13,078	8179
Population	High CVR, elevated TG levels, low HDL levels	High CVR, elevated TG levels, Diabetes
Treatment	DHA/EPA carboxylic acids (4 g/d) (Epanova^®^)	Icosapent-ethyl ester (4 g/d)
Placebo	Corn oil	Mineral oil
Follow-up Median	3.5 years	4.9 years
Primary Endpoint	Nonfatal stroke and MI, cardiovascular death, nonfatal MI, coronary revascularization or unstable angina	Nonfatal stroke and MI, cardiovascular death, coronary revascularization or unstable angina
95% CI of Primary Endpoint	0.99, 0.90–1.09	0.75, 0.68–0.83

Abbreviations: CI = Confidence Interval.

## Data Availability

No new data were created or analyzed in this study. Data sharing is not applicable to this review.
